# Successful recanalization of a calcified, collateral-poor LAD-CTO via repeat antegrade dissection and re-entry guided by intravascular ultrasound after prior subintimal plaque modification: a case report

**DOI:** 10.1186/s12872-026-05738-4

**Published:** 2026-03-23

**Authors:** Ziqi Li, Xuefei Mu, Bin Qi, Quanmin Jing, Yaling Han

**Affiliations:** Department of Cardiology, General Hospital of Northern Theater Command, 83 Wenhua Road, Shenyang, 110016 China

**Keywords:** Chronic total occlusion, Subintimal plaque modification, Intravascular ultrasound, Antegrade dissection re-entry

## Abstract

**Background:**

Chronic total occlusion (CTO) of the left anterior descending artery (LAD) with poor collateral circulation poses significant procedural challenges. Subintimal plaque modification (SPM) followed by repeat antegrade dissection and re-entry (ADR) under intravascular ultrasound (IVUS) guidance may enhance recanalization success.

**Case presentation:**

A 53-year-old male with prior non-ST-elevation myocardial infarction underwent failed initial percutaneous coronary intervention (PCI) for a stump-free, heavily calcified LAD-CTO with poor collaterals. After SPM, repeat angiography at 3 months revealed improved vessel architecture. IVUS-guided ADR was successfully performed, achieving full revascularization. At 4-year follow-up, the patient remained asymptomatic without major adverse cardiac events (MACE).

**Conclusion:**

In complex LAD-CTO lesions, SPM combined with IVUS-guided repeat ADR represents a viable bailout strategy, particularly when conventional antegrade/retrograde approaches fail.

**Supplementary Information:**

The online version contains supplementary material available at 10.1186/s12872-026-05738-4.

## Background

With continuous advances in interventional therapy for coronary artery disease, the overall success rate of percutaneous coronary intervention (PCI) has steadily improved; nevertheless, chronic total occlusion (CTO) lesions remain a formidable challenge, and procedural success remains suboptimal [1]. When conventional antegrade and retrograde strategies fail to re-enter the distal true lumen, subintimal plaque modification (SPM) can be employed as a bailout technique. At follow-up angiography, this strategy may restore antegrade flow or enhance the likelihood of successful repeat revascularization [2]. We herein report a case of unsuccessful antegrade dissection and re-entry (ADR) for a stump-free left anterior descending artery CTO (LAD-CTO) in which SPM was performed, followed by markedly different angiographic findings at 3-month re-evaluation. Under intravascular ultrasound (IVUS) guidance, ADR was subsequently applied and successfully recanalized the LAD-CTO.

## Case presentation

### Patient background

A 53-year-old male patient was admitted to our hospital on August 7, 2021, with a 3-month history of episodic chest pain. Three months prior, he had been diagnosed with acute non-ST-segment elevation myocardial infarction. Coronary angiography (CAG) revealed significant stenosis in the right coronary artery (RCA) and left circumflex (LCX) artery, with a CTO in the LAD artery. Stents were implanted in the RCA and LCX, but attempts to recanalize the LAD-CTO failed, and SPM was performed. The patient had a history of diabetes mellitus and a family history of coronary artery disease.

**Fig. 1:**
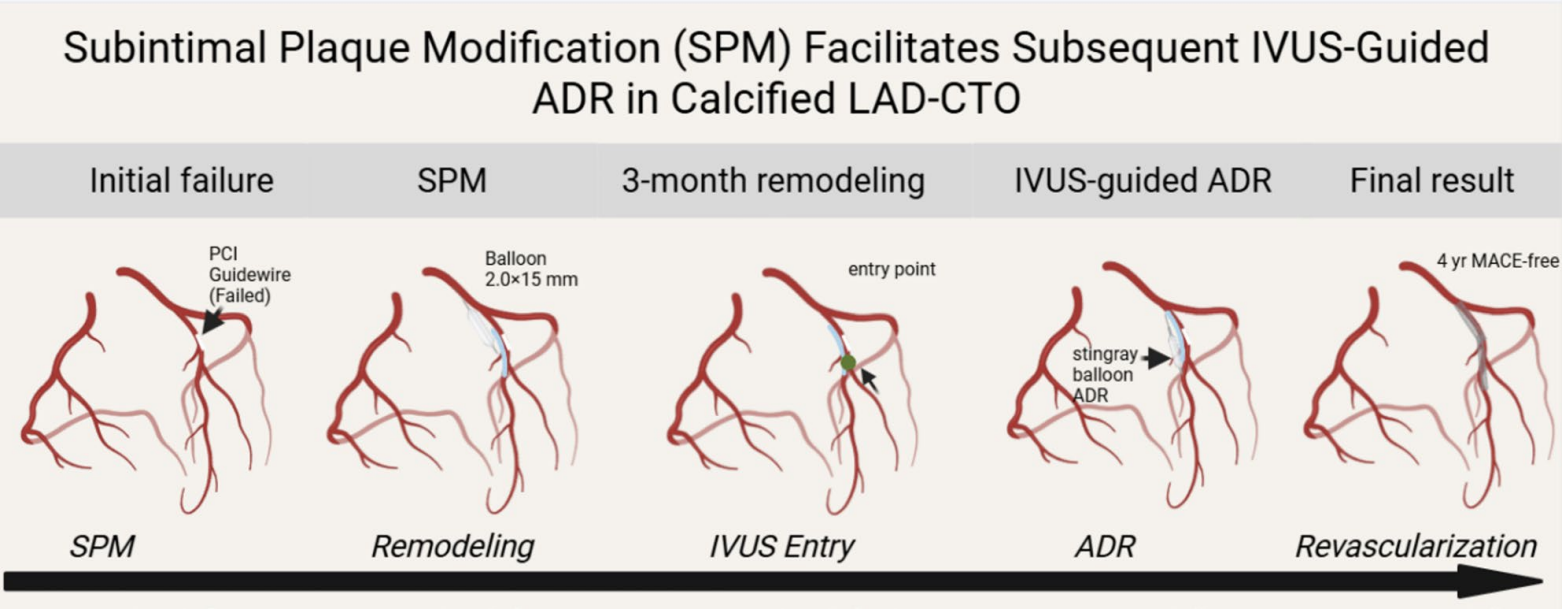
Technical flow chart：Subintimal Plaque Modification (SPM) Facilitates Subsequent IVUS-Guided ADR in Calcified LAD-CTO

**Fig. 2:**
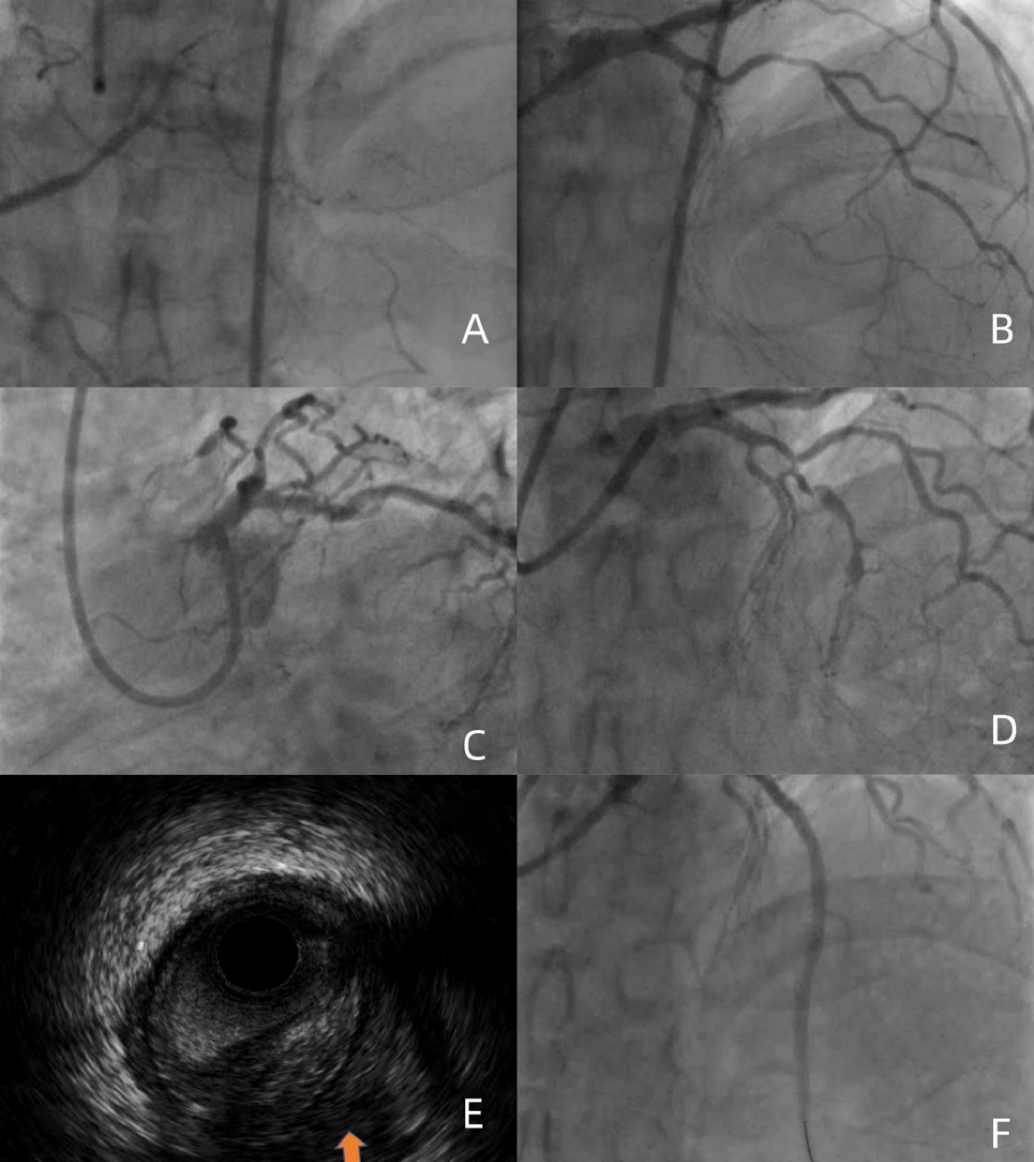
**A**: No visible collateral circulation from the RCA to the LAD; **B**: The LAD is occluded flush after the origin of the diagonal (D) and septal (S) branches; **C**-**D**: Follow-up angiography after SPM shows the apparent reappearance of an entry point and antegrade flow in the LAD; **E**: IVUS reveals multiple parallel lumens within the vessel architecture;(The orange arrow indicates the false lumen) **F**: Final result after stent implantation

**Fig. 3:**
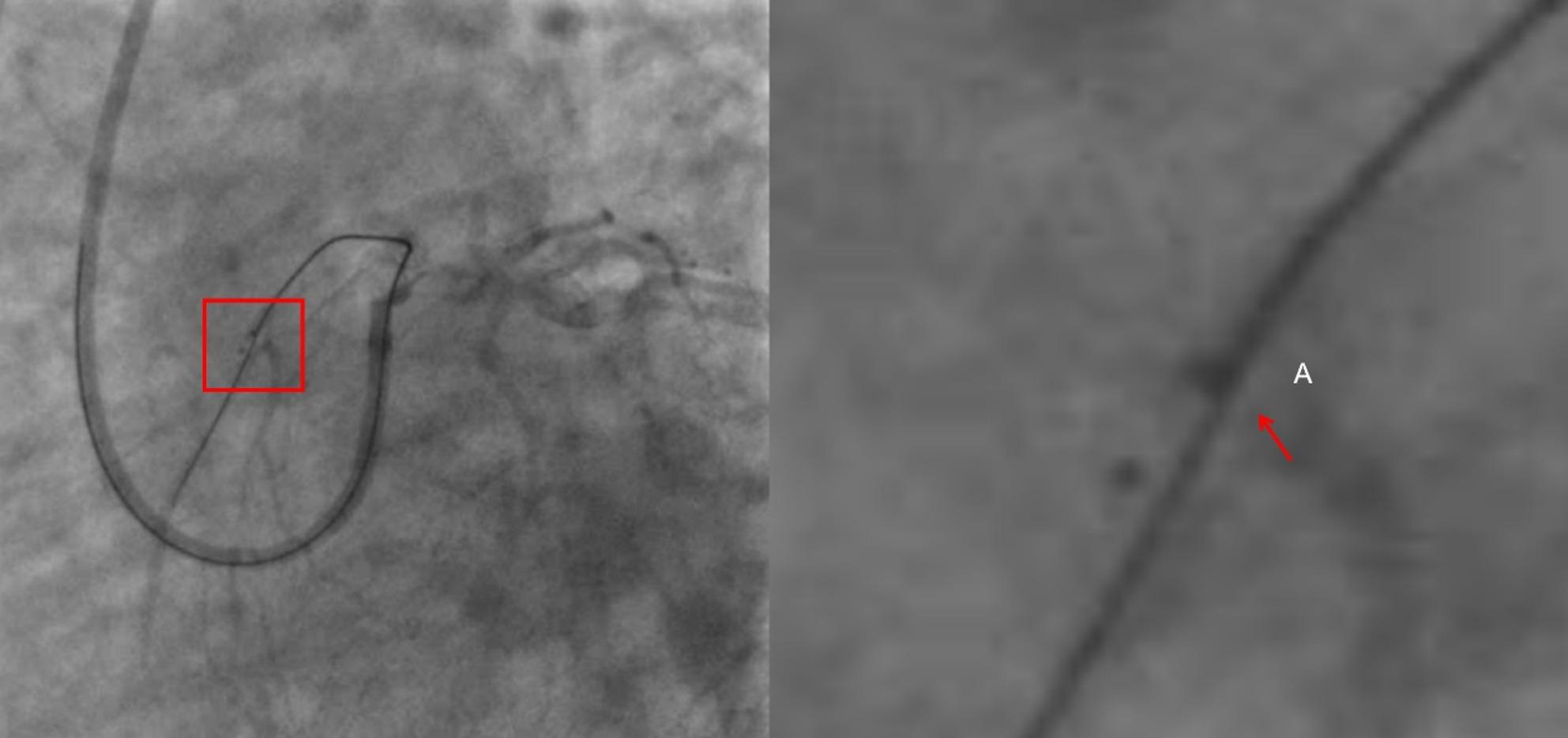
Spider-view angiogram showing the antegrade guidewire exiting at point A; right panel, magnified view of the wire exit site

This case presents a distinctive combination of anatomical challenges that are seldom reported together: (i) Rentrop-grade 0 collateral supply from both RCA and LCX, rendering a retrograde approach technically impossible; (ii) a flush, blunt ostial cap without any visible stump, increasing the risk of sub-intimal wire travel; and (iii) heavily calcified, ≥ 30 mm-long, ≥ 70° angulated occlusion—features that fulfil J-CTO ≥ 3 and PROGRESS-CTO ≥ 3 criteria for “extremely difficult” CTO. The deliberate use of IVUS-guided ADR after SPM has not been described previously for this specific phenotype. (Figure 1, 2, 3).

### Initial LAD-CTO Intervention (May 10, 2021)

CAG showed a CTO in the LAD artery, with no obvious collateral circulation from the RCA to the LAD. The initial strategy was antegrade, using a 7 F EBU 3.5 guiding catheter via the right femoral artery. Attempts with Fielder XT-R and Gaia 1ST wires failed. Parallel wire technique and knuckle wire technique were also unsuccessful, resulting in a hematoma. SPM was carried out with a 2.0 mm pre-dilation balloon advanced into the subintimal space of the LAD-CTO segment; the modified length fully encompassed the calcified portion. No peri-procedural complications—including coronary perforation, cardiac tamponade, or any other adverse events—were documented. During the initial attempt, after the guidewire entered the subintimal space, ADR was tried but failed because of an unclear entry point, rapid hematoma expansion, and poor visualization of the distal true lumen; consequently, the procedure was terminated and SPM was performed as a bailout.Consequently, the staged re-intervention was electively scheduled 90 days after the index SPM procedure [3].The formation of Coronary collateral circulation(CCC) in patients with CTO was determined by CAG.The CAG revealed that the collateral circulation from the RCA and LCX to the LAD was classified as Rentrop grade ≤ 1 [4] (Table 1). Additionally, the J-Channel score [5] was ≥ 3 (septal collateral channel grade CC-0 with retrograde tortuosity), indicating extremely poor collateral pathways and non-feasibility of the retrograde approach. Patients were stratified into three risk categories—easy, intermediate, and difficult—corresponding to summed scores of 0, 1–2, and ≥ 3, respectively.

**Table 1:** ༎Rentrop Coronary Collateral Grading Scale

Grade 0	No visible filling of any collateral channels;
Grade 1	Collateral filling of branches of the vessel to be dilated without any dye reaching the epicardial segment of that vessel;
Grade 2	Partial collateral filling of the epicardial segment of the vessel being dilated;
Grade 3	Complete collateral filling of the vessel being dilated.

### Follow-up angiography and re-attempt (August 9, 2021)

**Fig. 4:**
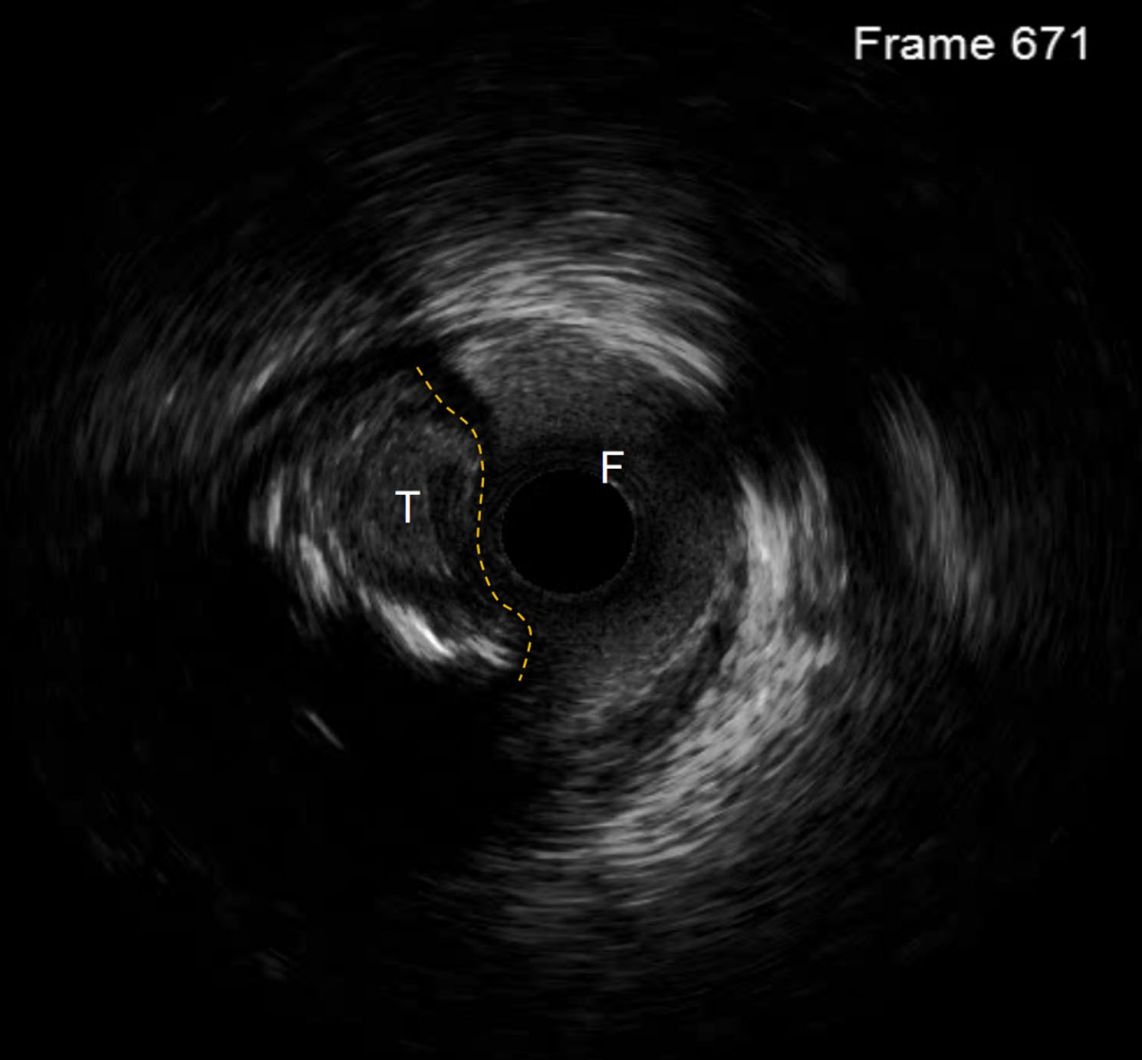
IVUS : true lumen (**A**), false lumen (**B**), yellow line indicates the intimal flap; the guidewire track documents ADR-based passage from true to false lumen

Follow-up angiography revealed an entry point and antegrade blood flow in the LAD, with parallel true and false lumens. The 3-month angiogram showed significant resolution of the previous hematoma, a newly tapered stump of the LAD, and improved visualization of the distal true lumen—changes attributed to vascular remodeling after SPM. These findings provided a favorable anatomic substrate for the second attempt. Initial attempts with Fielder XT-R and Gaia 3rd wires failed to re-enter the true lumen. IVUS was used to guide the procedure, confirming the true lumen entry via the diagonal branch. IVUS unequivocally distinguished the true lumen (three-layer echogenic signature: bright intima, dark media, bright adventitia) from the false lumen (single outer bright adventitial layer only), allowing us to orient the Stingray balloon so that its exit port faced the true lumen before Conquest-Pro puncture (Fig. 4).Following this, the knuckle wire technique was re-initiated. After multiple attempts, the Stingray balloon was employed to identify the true lumen, facilitating successful re-entry with the Conquest PRO 8–20 guidewire. Subsequent IVUS imaging-an extension of the sequence depicted in Video 1 A-unequivocally confirmed the guidewire tip position within the distal true lumen of the LAD. This was evidenced by the distinct visualization of the three-layer vessel wall architecture (intima, media, and adventitia) and the clear presence of an intimal flap tear, which further validated the guidewire’s successful passage into the true lumen (Video 3). Based on these IVUS findings, stenting was performed. The patient had no chest pain postoperatively and remained free of MACE during the 4-year follow-up.

## Discussion

In the context of complex CTO lesions, traditional antegrade and retrograde approaches may encounter significant limitations, particularly in reattempted procedures following prior failed recanalization attempts, where success rates remain suboptimal. Against this backdrop, SPM has emerged as a valuable adjunctive strategy in CTO interventions. SPM typically involves the creation of a controlled subintimal dissection using a knuckled guidewire followed by balloon dilation to modify the plaque architecture, with a planned reattempted intervention 2–3 months later. This approach has demonstrated favorable success rates in subsequent recanalization attempts [6–7].

For CTO lesions lacking a visible stump, procedural difficulty is markedly increased due to the absence of a clear entry point. In such cases, procedural success heavily relies on operator experience, as misidentification of the entry site can significantly compromise outcomes. Aggressive manipulation with stiff guidewires in pursuit of the true lumen may further escalate the risk of severe complications. IVUS can be instrumental in these scenarios by enabling precise identification of the true CTO entry via pullback imaging from adjacent branch vessels. Real-time IVUS guidance facilitates accurate puncture toward the true lumen, thereby enhancing procedural success rates [8].

This case strictly adhered to the stepwise CTO-PCI algorithm: after antegrade ADR failure the next default step was retrograde intervention. However, baseline angiography showed extremely poor collateral supply to the LAD (RCA and LCX septal branches CCC-0, epicardial J-Channel score ≥ 3), defining immediate retrograde impossibility. SPM was therefore undertaken as an “investment” bailout without delaying the patient. Repeat angiography 3 months later confirmed that collateral recruitment had still not occurred, so a second antegrade attempt was elected. The tapered tip, combined with a CTO length ≥ 30 mm and angulation ≥ 70°, made IVUS-guided ADR the ultimately effective adjunctive approach—particularly critical given the infeasibility of both conventional antegrade ADR and retrograde CART (controlled antegrade and retrograde tracking) at that stage.

Compared with previous SPM cohorts, our case demonstrated faster hematoma resolution—complete absorption within 90 days—and a newly tapered stump visible immediately after the short, 2-min SPM. Wire entry was followed at once by IVUS true-lumen seeking, turning an ambiguous-entry CTO into a single, predictable re-entry session. Current CTO-PCI algorithms accord IVUS-guided ADR a class-I indication when Stingray fails or hematoma obscures the target, and real-time three-layer imaging markedly increases first-puncture success. Thus, brief SPM coupled with IVUS-directed re-entry can safely conquer “extremely difficult” CTOs while avoiding the historical failure rate of blind sub-intimal re-entry.

Moreover, recent case reports have documented intramyocardial hematoma (IMH) as a rare but potentially catastrophic complication following CTO-PCI, particularly when aggressive antegrade manipulation or catheter pressure entrapment occurs [9]; this highlights the need for judicious use of high-pressure contrast injections and vigilant hemodynamic monitoring during complex CTO interventions.

In the application of ADR technique, meticulous control of intramural hematoma formation is essential. Maintaining the guidewire in close proximity to the vessel wall can facilitate smoother re-entry into the distal true lumen [10].

Studies have consistently demonstrated the safety and efficacy of knuckled guidewire techniques in both antegrade and retrograde CTO-PCI approaches [11]. The initiation site of knuckling is critical; positioning closer to the occlusion entry point correlates with higher success rates, provided that the correct entry is confirmed through multiplanar angiography and meticulous procedural patience.

In summary, this case report illustrates a comprehensive antegrade CTO recanalization strategy in the absence of retrograde feasibility. The procedural sequence encompasses progressive escalation of antegrade guidewire techniques, parallel-wire strategies, ADR utilization, and optimal timing for knuckled guidewire deployment. Furthermore, in instances of antegrade technical failure, the case effectively highlights the advantages of SPM combined with IVUS guidance in stump-free CTO lesions. These insights hold significant educational value and warrant broader dissemination in clinical practice.

## Conclusion

This case illustrates that in stump-free, heavily calcified LAD-CTOs with poor collaterals, initial SPM substantially remodels the vessel architecture, and subsequent IVUS-guided repeat ADR constitutes a safe, reproducible bailout technique for durable revascularization with low long-term MACE, warranting broader clinical implementation.

## Supplementary Information

Supplementary Material 1.

Supplementary Material 2.

Supplementary Material 3.

## Data Availability

The data that support the findings of this study are available from the corresponding author upon reasonable request.

## Abbreviations

Abbreviation: Meaning
ADR: Antegrade dissection and re-entry
CAG: Coronary angiography
CTO: Chronic total occlusion
IVUS: Intravascular ultrasound
LAD: Left anterior descending artery
LCX: Left circumflex artery
MACE: Major adverse cardiac events
PCI: Percutaneous coronary intervention
RCA: Right coronary artery
SPM: Subintimal plaque modification
